# Resilience in shock and swim stress models of depression

**DOI:** 10.3389/fnbeh.2013.00014

**Published:** 2013-02-28

**Authors:** Robert C. Drugan, John P. Christianson, Timothy A. Warner, Stephen Kent

**Affiliations:** ^1^Department of Psychology, University of New HampshireDurham, NH, USA; ^2^Department of Psychology and Neuroscience, University of Colorado at BoulderBoulder, CO, USA; ^3^School of Psychological Science, LaTrobe UniversityBundoora, VIC, Australia

**Keywords:** shock and swim stress, GABA_A_ receptor, neurosteroids, resilience, ultrasonic vocalizations

## Abstract

Experimental models of depression often entail exposing a rodent to a stressor and subsequently characterizing changes in learning and anhedonia, which may reflect symptoms of human depression. Importantly, not all people, and not all laboratory rats, exposed to stressors develop depressed behavior; these “resilient” individuals are the focus of our review. Herein we describe research from the “learned helplessness” and “intermittent swim stress” (ISS) models of depression in which rats that were allowed to control the offset of the aversive stimulus with a behavioral response, and in a subset of rats that were not allowed to control the stressor that appeared to be behaviorally and neurochemically similar to rats that were either naive to stress or had controllability over the stressor. For example, rats exposed to inescapable tailshock, but do not develop learned helplessness, exhibit altered sensitivity to the behavioral effects of GABA_A_ receptor antagonists and reduced *in vitro* benzodiazepine receptor ligand binding. This pattern suggested that resilience might involve activation of an endogenous benzodiazepine-like compound, possibly an allostatic modulator of the GABA_A_ receptor like allopregnanolone. From the ISS model, we have observed in resilient rats protection from stressor-induced glucocorticoid increases and immune activation. In order to identify the neural mediators of these correlates of resilience, non-invasive measures are needed to predict the resilient or vulnerable phenotype prior to analysis of neural endpoints. To this end, we found that ultrasonic vocalizations (USVs) appear to predict the resilient phenotype in the ISS paradigm. We propose that combining non-invasive predictive measures, such as USVs with biological endpoint measures, will facilitate future research into the neural correlates of resilience.

Depression is a widespread disorder resulting in significant suffering for the patient and their families (Nestler et al., 2002; Knol et al., 2006). Although great strides have been made in the last 50 years toward improving antidepressant pharmacotherapies (Berton and Nestler, 2006; Drevets et al., 2008), fewer than one half of the people prescribed antidepressant drugs respond favorably to treatment and remain refractory (Southwick et al., 2005; Berton and Nestler, 2006). Exposure to stressors over the lifespan is a good predictor of risk for depression, and the prevailing view is that depressed mood is the result of an interaction between stressors and genetic factors (Caspi et al., 2003, 2010; Southwick et al., 2005). However, understanding an individual's stress history and genetic risk is not sufficient for understanding vulnerability to depression, as many people experience chronic or severe stress without ever developing major depression. Thus, many individuals are resilient to depression. The goal of the work reviewed here was to identify behavioral and neural characteristics of resilience with the hope of illuminating previously unappreciated candidates for therapeutic drug development and preventative therapies.

It is important to note at the outset that we conceptualize resilience broadly. For instance, an individual may appear to be resilient because of previous experiences that rendered the individual *resistant* to the stressor's consequences, because of an inherent capacity to *recuperate* after trauma, or because of their ability to *mitigate* the physiological or psychological consequences of a stressor by implementing effective coping strategies (for further discussion see Fleshner et al., 2011). Our research into the neural correlates of resilience began in the early 1980's in the laboratory of Dr. Steven Maier using the “learned helplessness” model of depression (Maier et al., 1973; Maier and Seligman, 1976). Early research by Maier and Seligman (1976) demonstrated that the controllability of a stressor was one of the most important predictors of stressor consequences on behavior. Initial studies focused on instrumental shuttle escape learning in dogs and rats (Overmier and Seligman, 1967; Maier et al., 1973), but have since expanded to include activity measures (Jackson et al., 1978; Drugan and Maier, 1983), food competition dominance (Rapaport and Maier, 1978), rewarding effects of drugs (Will et al., 1998), and social behavior (Short and Maier, 1993; Christianson et al., 2009). In each of these cases, exposure to unpredictable and inescapable shocks (inescapable stress, IS) resulted in behavioral changes that reflect aspects of anxiety and depression. However, if the shocks were escapable (escapable stress, ES) by means of performing a behavioral wheel-turn response, then behavior appeared normal in subsequent tests. Thus, the controllability of the stressor determined whether the subject would appear resilient or vulnerable. These, “stressor controllability effects” have been reviewed elsewhere (Maier and Watkins, 2005, 2010).

## Stressor controllability and stress-induced analgesia

**Figure 1:**
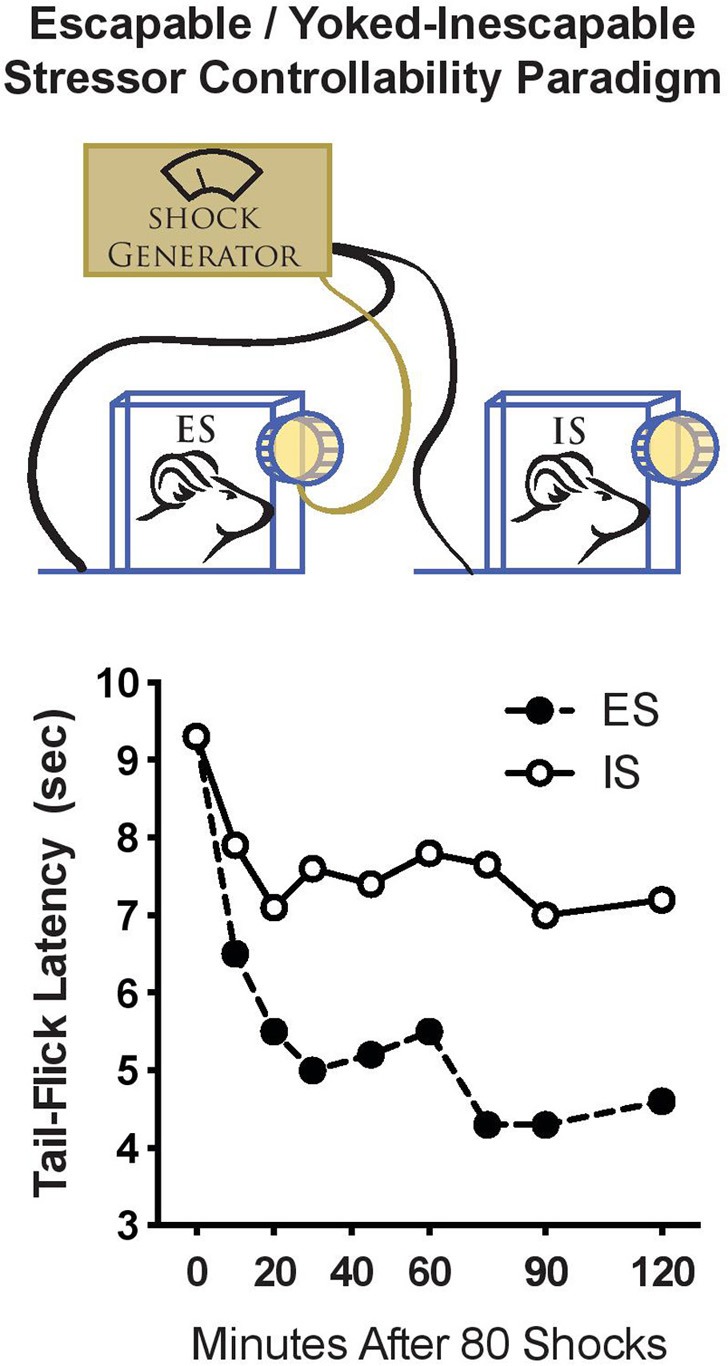
**Stressor controllability and resilience to tailshock. Top:** A schematic diagram of the stressor controllability experiment. Rats are assigned to either escapable stress (ES), inescapable stress (IS), or no stress. Rats in the ES group receive a series (usually 80–100 trials) of tailshocks while restrained in a chamber with a wheel that is connected to a controlling computer. Rats in the ES group may escape from shocks by turning the wheel. Rats in the IS group are “yoked” to the ES rat such that shock is terminated for the IS when the wheel turn response is made. The wheel in the IS chamber is not connected to the computer, so these rats do not have control over shock onset or offset. Importantly, each rat in the pair receives exactly equal tailshock. **Bottom:** Stressor controllability and analgesia. Rats were exposed to 80 trials of ES or IS, and pain tolerance was quantified using the tail-flick reflex to heat applied to the tail at various times after stress. Both ES and IS rats displayed analgesia immediately after stress. However, analgesia persisted for 2 h after IS while ES rats recovered (adapted from Drugan et al., 1985a).

Studies of stressor controllability typically employ a “triadic design,” which permits the experimenter to manipulate only the variable of control while the amount and number of shocks are equal (Figure 1). At the time we began studying stressor controllability, the major focus was on identifying the mechanisms that caused learned helplessness; specifically, the shuttle escape learning deficit that occurs only after IS exposure. It was hypothesized that exposure to IS caused a change in central analgesia systems, such that subsequent exposure to footshocks in the shuttle escape task would not be sufficiently motivating because of an enhanced analgesia (see Maier, 1986 for a review of behavioral stress-induced analgesia studies). Interestingly, we found a very different activation of pain inhibition systems during the stress experience depending on whether the rats experienced ES vs. IS. IS induced a long-lasting analgesia mediated by endogenous opioids. Importantly, ES also induced analgesia, but it was much shorter and independent of endogenous opioids (Figure 1; Maier et al., 1982; Drugan et al., 1985a). This indicated that stressor controllability determined what type of pain inhibition systems were activated in response to stress. It was hypothesized that the non-opioid form of analgesia observed as a result of ES would enable the coping behavior, whereas the opioid analgesia observed after IS would inhibit behavioral responses (Maier, 1986).

## Stressor controllability and GABA_A_

We were intrigued about the identity of this coping-induced analgesia that could orchestrate such a differential reaction to the stress. Mineka and colleagues (1984) noted that ES vs. IS rats exhibited very different behaviors in between shocks. In fact, they reported greater fear responses associated with the IS vs. ES context. Thus, we tested whether the stressor-induced analgesia was dependent upon fear or anxiety experienced during shock exposure. Indeed, administration of the anxiolytic benzodiazepine, chlordiazepoxide (Librium), before IS prevented the development of learned helplessness and long-lasting analgesia (Drugan et al., 1984). Conversely, administration of the anxiogenic β-carboline, FG 7142, in lieu of IS exposure, produced a learned helplessness-like shuttlebox escape deficit (Drugan et al., 1985b). Together these studies provided evidence that mitigating anxiety might be necessary for the prevention of learned helplessness effects that occurred in ES rats.

**Figure 2:**
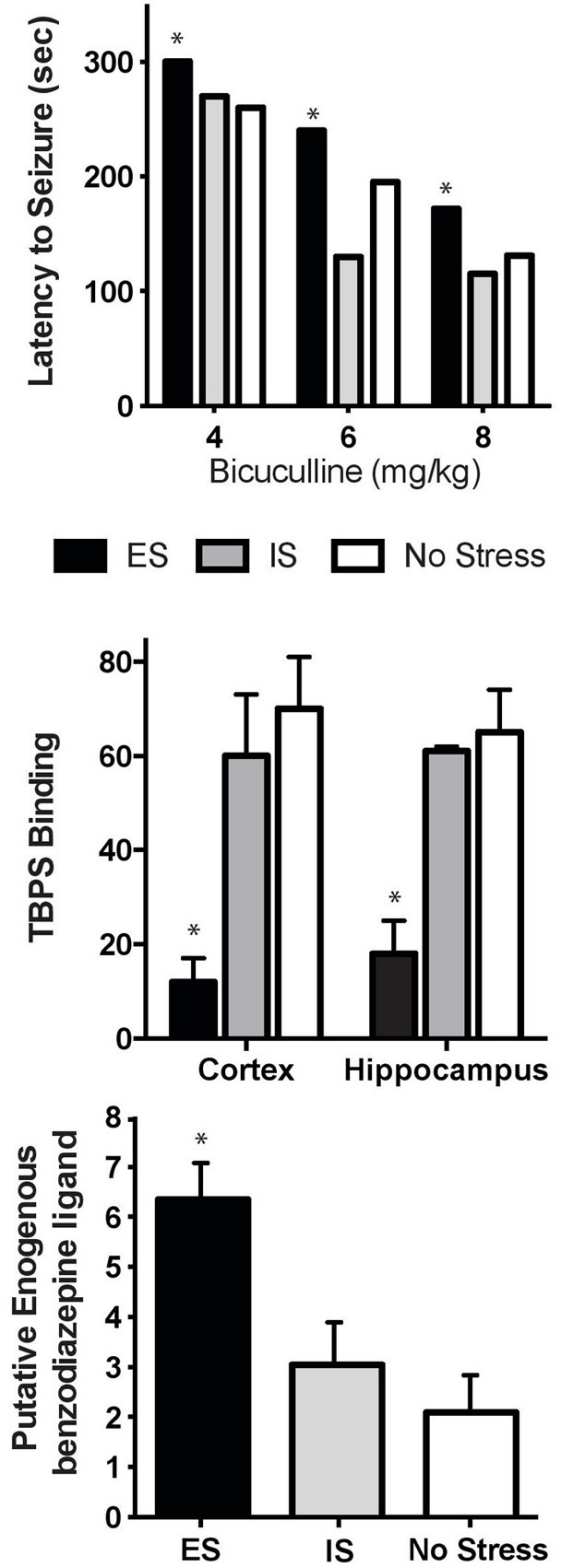
**Stressor Controllability and Central Benzodiazapine/GABA_A_ receptor. Top:** Rats were exposed to ES, IS, or no stress, and then administered increasing doses of bicuculline 2 h after stress. Rats with prior ES appeared to be protected from seizures with a significant delay in the onset of clonus seizure symptoms compared to unstressed rats [ES had a significantly longer latency compared to no stress controls (^*^p < 0.05). Adapted from Drugan et al., 1985c]. **Middle:** Mean (+SEM) [^35^S]TBPS receptor binding (2 nM, fmol/mg protein brain tissue) 2 h after stress. Exposure to ES significantly reduced competitive binding of the benzodiazepine receptor ligand (^*^p < 0.05, adapted from Drugan et al., 1994). **Bottom:** Mean (+SEM) brain levels of benzodiazepine receptor agonist molecules (ng/mg brain tissue) 2 h after stress. Prior ES significantly increased levels of endogenous benzodiazepine receptor ligands (^*^p < 0.05, adapted from Drugan et al., 1994).

Since benzodiazepines and β-carbolines act at the GABA_A_ receptor (Braestrup et al., 1980; Paul and Skolnick, 1982), this was an obvious place to start our systematic investigation. The hypothesis to be tested was that ES would facilitate GABAergic tone (mimicking an anxiolytic agent), while IS would interfere with GABAergic tone (similar to an anxiogenic agent). We first utilized bicuculline-induced seizures as a behavioral assay of GABA_A_ receptor function after ES or IS. If ES increased GABA_A_ function, then significantly greater concentrations of the GABA_A_ antagonist would be required to induce seizure and vice versa for IS. This is precisely what occurred with rats exposed to ES protected from either bicuculline or picrotoxin-induced seizures, while IS rats showed an increased susceptibility to seizure (Figure 2; Drugan et al., 1985c, 1994). The functional significance of this change in GABAergic sensitivity was revealed in that stressor controllability altered the hypnotic and ataxic effects of several central nervous system depressants. More specifically, immediately or 2 h following inescapable shock stress, we observed an enhanced reactivity to both ethanol and midazolam compared to non-shocked controls. However, exposure to escapable shock did not change the reactivity to these minor tranquilizers (Drugan et al., 1992, 1996). Thus, it appeared that providing rats with a coping mechanism altered central GABAergic function. Protection against picrotoxin-induced convulsions, and its association with brain benzodiazepine receptor occupancy, has been well-established (Duka et al., 1979; Braestrup et al., 1982; Mennini and Garattini, 1982; Paul et al., 1982). It was hypothesized that ES would stimulate the release of endogenous ligands for the benzodiazepine binding site on the GABA_A_ receptor and, thereby, result in allosteric changes to the GABA_A_ site. Thus, after ES, fewer binding sites would be available for the experimenter-administered bicuculline. Indeed, *in vitro* radioligand binding assays of the GABA_A_ receptor in rats following ES or IS revealed that rats exposed to ES exhibited decreased [^35^S]T-butylbicyclophosphorotionate (TBPS) binding to the picrotoxin site on the GABA_A_ receptor in cortex and hippocampus tissue when compared to IS rats and controls (Drugan et al., 1994).

As with clinical depression, not all rats exposed to IS develop learned helplessness. More specifically, a portion of rats initially exposed to IS learn to escape in the shuttlebox test. Rats were exposed to IS and then tested for shuttle escape performance 24 h later. The shuttle escape task requires that the rat shuttle from one side of the apparatus to the other to terminate the footshock. Rats that exhibit learned helplessness typically “fail” this task, and subsequently the experimenter terminates the shocks, typically after 30 s. An arbitrary criterion was established to split rats into “fail” and “learn” groups based on a median split in average escape latencies. Thus, two non-overlapping distributions of rats were identified, and this pattern has been reported by numerous groups (Chi et al., 1989; Drugan et al., 1989a,b; McIntosh and Gonzalez-Lima, 1994; Minor et al., 1994; Koen et al., 2005). Furthermore, the behavioral profiles of these rats remained the same when challenged with an IS 2 or 4 weeks later, and were subsequently tested for shuttle escape performance. This indicated that this initial stress reactivity was a rather stable trait (Drugan et al., 1989b). We then tested the GABA_A_ binding in rats that were divided into “failers” and “learners” as above. The brains of the group that learned (e.g., were resilient) showed an increased binding of [^3^H] muscimol to the GABA_A_ receptor in cerebral cortex, but a reduction in [^35^S]TBPS binding to the benzodiazepine binding site on the GABA_A_ receptor (Drugan et al., 1993). These findings, and those from rats exposed to ES, suggested that the resilience to tailshock involved activation of an endogenous benzodiazepine-like ligand. To our knowledge, this was the first evidence that ES, or resilience to IS, activated endogenous neural machinery that could mitigate the anxiety and analgesia evoking consequences of shock exposure *per se*.

## Stressor controllability and endogenous anxiolytic factors

The search for endogenous ligands for the benzodiazepine/GABA_A_ receptor began in the late 1970's and has continued into the present. Putative endogenous ligands include purines (Skolnick et al., 1978, 1980), hemoglobin metabolites (Ruscito and Harrison, 2003), diazepam binding inhibitor (DBI; Costa et al., 1994), octadecaneuropeptide (ODN; Do-Rego et al., 2001), neuroactive steroids such as 3 alpha, 5 alpha-tetrahydrodeoxycorticosterone (THDOC; Majewska et al., 1986), and brain-derived neurosteroids such as pregnenolone (Jung-Testas et al., 1989). Although the ES-induced endogenous ligand remains unknown, there is evidence in support of neurosteroids. First, the effects on benzodiazepine/GABA_A_ binding reviewed above occur in adrenalectomized rats, indicating that the endogenous anxiolytic factor is not a product of the adrenal gland (i.e., a neuroactive steroid such as THDOC; Drugan et al., 1993). Second, evidence exists from a study in which we observed a proactive interference of ES on a subsequent spatial memory task, an effect that depended on neurosteroid synthesis (Healy and Drugan, 1996). There is clear evidence that *de novo* steroid synthesis can occur in the brain (Jung-Testas et al., 1989). Pregnenolone is the primary precursor of steroid hormone biosynthesis in adrenal tissue (Hechter et al., 1951; Brown et al., 1979; Jung-Testas et al., 1989), yet this substance is found in the brain of rats or monkeys with either surgical or pharmacological removal of peripheral steroid secretion (Robel et al., 1987). Given this information, we hypothesized that both ES exposure as well as resistance to IS effects may be the result of the release of a positive modulatory, brain derived neurosteroid. One such candidate is the A-ring-reduced metabolite of progesterone, 3 alpha-hydroxy-5 alpha-pregnan-20-one (allopregnanolone). Importantly, this substance inhibits [^35^S]TBPS binding (Gee et al., 1987) and has anticonvulsant (Belelli et al., 1989), and anxiolytic properties (Crawley et al., 1986). Finally, stress-induced increases of alloprenanolone have been observed for 1 h following 5–10 min ambient swim stress in sham as well as adrenalectomized rats (Purdy et al., 1991). Allopregnanolone represents a novel target for therapeutic drug development. Testing the hypothesis that stress-induced synthesis and release of allopregnanolone contributes to resilience is the focus of future research.

## Probing the generality of stressor controllability effects in a novel intermittent swim stress paradigm

The tenacious pursuit of neural mechanisms mediating stressor controllability effects by Maier and colleagues has provided very exciting insight into the pathophysiology of stress related disorders and novel treatments. However, the vast majority of reported research into stressor controllability has utilized tailshock as a stressor. Although tailshock has some advantages as a stimulus (i.e., it is aversive without producing tissue damage, it can be precisely administered, rats readily learn to escape it, and it does not lead to habituation) one might wonder if the results from studies of controllability and resilience to tailshock generalize to stressor exposure *per se.* To this end, we developed an intermittent swim-stress (ISS) version of the stressor controllability paradigm in which rats are exposed to brief, unpredictable forced swims that can be terminated in the escapable swim group by pressing a lever hanging in the middle of the swim chamber. ISS is conducted in a plastic chamber with a false floor that can be raised and lowered into a tank of water; the idea was to create an intermittent version of the forced swim test (FST) that is widely used [Porsolt et al., 1977, 1979; see Brown et al. (2001), or Drugan et al. (2005) for a photo of the ISS apparatus], with the only difference being the temperature of the water between paradigms.

As with shock, the controllability of swim determines the behavioral outcome. Twenty-four hours after stress, rats exposed to inescapable, but not escapable ISS, display increased immobility during a 5 min FST (Drugan et al., 2005). We have also reported a learned helplessness-like, escape learning deficit in rats exposed to inescapable ISS in something we termed the “swim escape test” (SET). The SET places rats in the swim apparatus with a lever positioned at the surface of the water during a trial. The rats are required to press the lever once (fixed-ratio; FR-1) during the first five trials and twice (FR-2) during the subsequent trials in order to escape from the forced swim. The instrumental requirements were developed to be identical to the learned helplessness shuttlebox escape test (Maier et al., 1973). Again, similar to inescapable tailshock, some of the rats previously exposed to ISS fail to learn the escape response, while others do learn and appear to be resilient (Christianson and Drugan, 2005). We hope to extend the generality of findings from the learned helplessness, tailshock stress paradigm to stress *per se* by determining whether the neural substrates such as the medial prefrontal cortex, found to be important in the work of Maier and colleagues (see Maier and Watkins, 2010 for review) apply to controllability of swim stress. Rearing and housing conditions also play a role in subsequent resilience and vulnerability with early weaning and isolation, but not maternal separation, predicting more depressive-like behavior (i.e., increased mean swim time in the SET). Similarly, maternal contributions also play a role; pups from dams that displayed increased anxiety-like behavior had longer swim times in the SET and were more likely to be classified as vulnerable (Stiller et al., 2011). Thus, the ISS model provides the same empirical features as the tailshock paradigm: the behavioral consequences depend on stressor controllability and rats can be identified as stress-resilient or -vulnerable based on the performance of an escape test.

## Immune correlates of resilience and vulnerability in the intermittent swim stress paradigm

Since conducting the pharmacology and behavioral studies discussed above in the shock paradigm, our interests and technologies have evolved. With the ISS model, we became interested in examining the consequences of stressor exposure on immune endpoints as inflammatory processes are implicated in the pathophysiology of depression (Raison et al., 2006; Dantzer et al., 2008). Using a median split in SET performance to identify resilient and vulnerable samples after ISS, stress vulnerable rats exhibited increased post-SET plasma corticosterone (CORT) concentrations, and enhanced T-cell proliferation in response to conconavalin-A (Con-A) compared to stress resilient rats (Levay et al., 2006; Stiller et al., 2011). Because rats that learn to escape would experience significantly less exposure to swim than those that failed, and accordingly less exposure to cold water, core body temperature was assessed in rats following the SET. Not surprisingly, rats that exhibited poor escape learning and long escape latencies (i.e., vulnerable) exhibited greater hypothermia than rats with good escape learning and short escape latencies (i.e., resilient). This difference amounted to an additional 7.4 min of total swim time (11.7 vs. 4.3 min), which led to a 2.0°C decrease in body temperature (Levay et al., 2006). Unfortunately, the results from endocrine and immune measures (after SET) are confounded by both hypothermia and differential exposure to swim.

Although these ISS results are encouraging, there is a potential confound in that the groups are typically measured following a subsequent test, which is a stressor itself (e.g., social interaction, social defeat, shuttlebox performance, and SET). The tests themselves may change brain chemistry and mask the “true” changes due to stress resistance interfering with the identification of neural processes underlying resilience. In addition, the subjects in tests, such as shuttlebox escape, receive unequal amounts of stress (e.g., less shock in subjects that learn and more shock in those that fail). Similarly, in the SET, rats show differential escape behavior, which results in a different amount of swim stress exposure among all rats prior to extraction of the brain to look at neural changes. For example, this makes any brain changes observed following these tests uninterpretable, because of the potential confound of differential stress exposure during the test causing the differences and not stress resistance *per se*. This drawback is not unique to the ISS paradigm. Many of the strategies used in the field to investigate resilience involve experimentally manipulating the environment in some fashion and a behavioral assay to identify resilient vs. vulnerable populations (see Russo et al., 2012, for recent review). In many cases the assay for vulnerability, in our case the SET or the shuttlebox test, introduce a confounding variable that make subsequent analyses of brain, immune, or other endpoints difficult to interpret. However, in the Drugan et al. (1993) study, the GABA_A_ receptor changes were observed in resilient rats that were given 3–4× the number of escape trials in an effort to equilibrate shock exposure to the vulnerable rats. Nonetheless, the pattern of the shock exposure still differed between vulnerable (longer duration shocks on each trial) and resilient (shorter duration shocks on each trial), and this still may influence brain changes.

## Ultrasonic vocalizations as a correlate of resilience

In order to conduct post stress analyses on resilient and vulnerable populations, one would need a tool that reliably predicts performance in assays like the SET and shuttlebox that is both non-stressful and non-invasive. To this end, we began to record ultrasonic vocalizations (USVs) emitted by rats during ISS exposure. USVs are emitted by rats following significant environmental events and can be used as an “on-line” measure of emotional status (Knutson et al., 2002). USVs have been used to indicate exposure to cold (Blumberg and Stolba, 1996), or as a correlate of fear behavior in developmental studies (Brunelli and Hofer, 1996; Dichter et al., 1996). USVs are categorized based on a combination of frequency and duration dimensions (Litvin et al., 2007). Adult rats emit “22-kHz” and “50-kHz” USVs (Brudzynski et al., 1993; Panksepp et al., 1998). Fifty-kilohertz calls are associated with positive or approach situations (Knutson et al., 1998; Panksepp et al., 1998), whereas 22-kHz USVs occur in response to aversive stimuli (Tonoue et al., 1986; Blanchard et al., 1991; Miczek et al., 1991, 1995; Knapp and Pohorecky, 1995; Brudzynski, 2001; Swiergiel et al., 2007) and during defensive/submissive behavior (Thomas et al., 1983; van der Poel and Miczek, 1991). Thus, 22-kHz USVs tend to be emitted during distress and correlate with negative affect (Brudzynski et al., 1993; Panksepp et al., 1998), although exceptions have been noted (Barfield and Geyer, 1972, 1975; van der Poel and Miczek, 1991). However, a recent finding suggested that USVs might be a predictor of stress resilience. Jelen et al. (2003) reported that rats emitted 22 kHz USVs in the presence of a safety signal rather than a danger signal. This laboratory investigation confirmed observations in the field where rodents emitted USVs when they were in a position of safety, yet observed the approach of a predator (Litvin et al., 2007).

**Figure 3:**
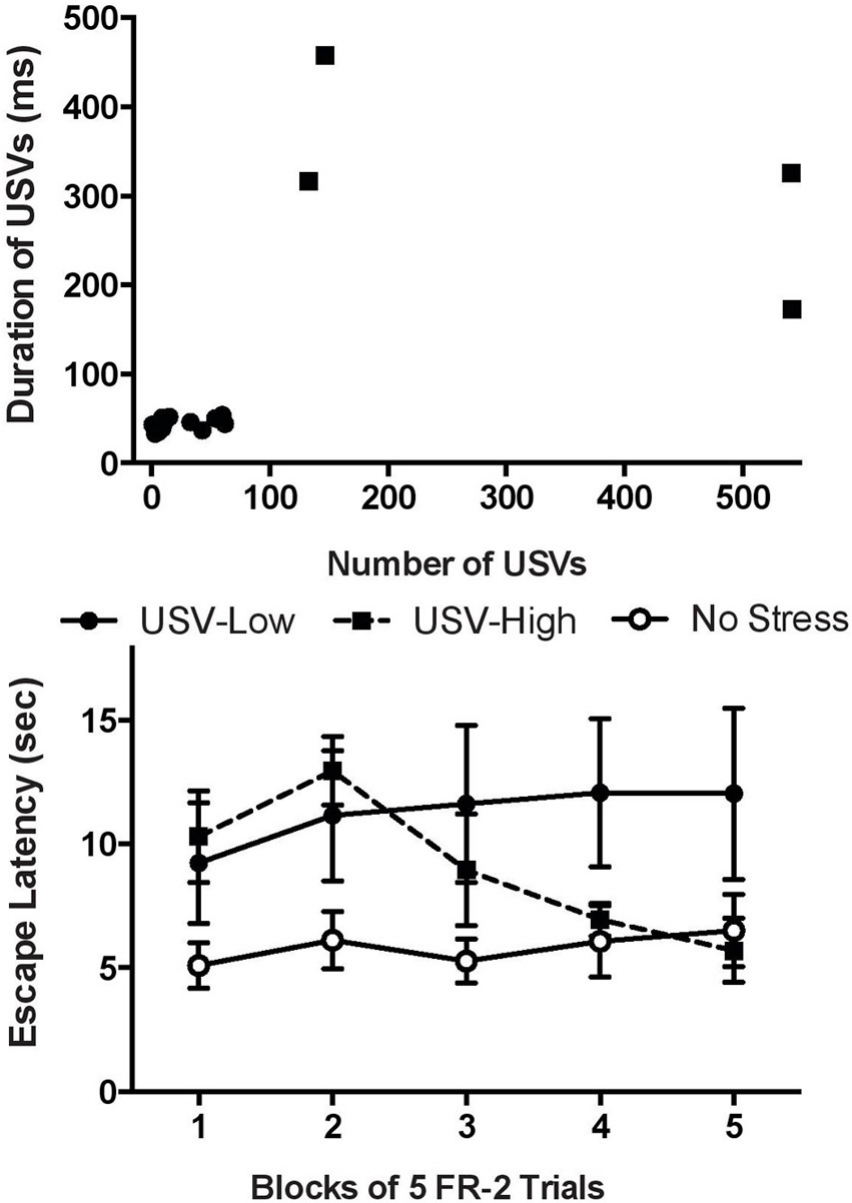
**Predicting resilience in the Swim Stress Paradigm. Top:** A scatter plot characterizing the ultrasonic vocalizations (USVs) of the rats in the swim stress group recorded during stress exposure. Rats were categorized as USV-High rats (black squares) or USV-Low (black circles). USV-High rats produced a large number of long-duration vocalizations while USV-Low rats emitted few, short-duration vocalizations. Plotted are the number of ultrasonic vocalizations vs. the average duration of the vocalizations for each rat. **Bottom:** Mean (±SEM) time to escape in the swim escape test (SET). Rats in the USV-Low group displayed a detriment in learning while the USV-High group appeared resilient and performed similarly to the no stress control group. The no stress group was exposed to the swim stress apparatus, but was never exposed to swim (adapted from Drugan et al., 2009).

Given this information, USVs were recorded throughout ISS exposure and two distinct populations of rats were identified. One group produced many long duration USVs, while another made very few USVs with shorter durations (Figure 3; Drugan et al., 2009). Upon subsequent exposure to the SET, rats in the first group appeared to be resilient with good escape learning, while the rats in the later group did not learn to escape (Figure 3; Drugan et al., 2009). Thus, the high number and long-duration USVs made during ISS appear to be a good predictor of resilience in the SET. An important next step in this research line is to determine if USVs also predict resilience in other behavior endpoints such as forced swim or anxiety behaviors. Consistent with our initial report, several recent studies have used USVs to predict behavior in a drug self-administration paradigm (Maier et al., 2012; Meyer et al., 2012). The data encourage continued assessment of USVs to establish this as a useful tool to forecast subsequent stress reactivity. As noted, such a tool will eliminate the confounding effect of post-stress tests used to behaviorally identify resilient and vulnerable populations. By using USVs as a predictor, the experimenter can be sure that both resilient and vulnerable populations have received identical stressor exposure.

## Conclusions and future directions

The results of the research reviewed support a few points that should inform continued research into the neural correlates of resilience. Foremost is the importance of understanding the confounding influence of the behavioral test employed to identify resilient vs. vulnerable populations of subjects. Some of the work we have reviewed must be considered in light of these confounds. The behavioral and biological endpoints quantified were assessed after rats performed differentially in tasks that would result in different exposure to stressful stimuli—such as footshock or forced swimming. Our work, in concert with that of others, has built support for emitted USVs as a way to forecast resilient populations so that neural or physiological endpoint measures may be conducted without introducing additional stressors—a non-invasive measure of stress reactivity. Also important is the possible role of endogenous modulators of the GABA_A_ receptor, such as allopregnanolone in stress resilience. Questions remain regarding the central sites of synthesis and action of this neurosteroid during ES, whether behavioral correlates of resilience (e.g., USVs) are causally linked to neurosteroid actions, and whether stress inhibitory neural circuits interact with the neural loci that support production of USVs. These are the focus of ongoing research and should broaden our understanding of resilience and provide new avenues for antidepressant development.

### Conflict of interest statement

The authors declare that the research was conducted in the absence of any commercial or financial relationships that could be construed as a potential conflict of interest.
